# Online Guided Self-help Cognitive Behavioral Therapy With Exposure to Anxiety and Problem Solving in Type 1 Diabetes Mellitus: Case Study

**DOI:** 10.2196/32950

**Published:** 2022-07-13

**Authors:** Dorian Kern, Brjánn Ljótsson, Marianne Bonnert, Nils Lindefors, Martin Kraepelien

**Affiliations:** 1 Centre for Psychiatry Research Department of Clinical Neuroscience Karolinska Institutet & Stockholm Healthcare Services Stockholm Sweden; 2 Division of Psychology Department of Clinical Neuroscience Karolinska Institutet Stockholm Sweden; 3 Department of Medical Epidemiology and Biostatistics Karolinska Institutet Stockholm Sweden

**Keywords:** fear of hypoglycemia, problem solving, cognitive behavioral therapy, psychology, self-help, diabetes, type 1 diabetes mellitus, high blood glucose

## Abstract

**Background:**

Type 1 diabetes mellitus (T1DM) is dependent on self-care to avoid short- and long-term complications. There are several problem areas in diabetes that could be addressed by psychological interventions, such as suboptimal problem-solving strategies and fear of hypoglycemia. There is empirical support for a few psychological interventions, most often cognitive behavioral therapy, with various treatment aims. However, these interventions are largely unavailable in regular diabetes health care. Online guided self-help cognitive behavioral therapy could help achieve greater outreach.

**Objective:**

We tested a manualized treatment in the early stage for further development, with the long-term aim to increase access to care. The purpose of this report was to show the potential of this newly developed online intervention by describing 2 illustrative cases.

**Methods:**

An online guided self-help cognitive behavioral therapy protocol featuring problem solving and exposure was developed. The treatment was administered from a secure online platform and lasted for 8 weeks. Case 1 was a male participant. He had a number of diabetes-related complications and was worried about his future. He reported that he had a general idea that he needed to change his lifestyle but found it difficult to get started. Case 2 was a female participant. She had fear of hypoglycemia and unhelpful avoidance behaviors. She kept her blood glucose levels unhealthily high in order to prevent hypoglycemic episodes. Furthermore, she avoided contact with diabetes health care.

**Results:**

The 2 participants showed clinically significant improvements in their most relevant problem areas. In case 1, the participant’s blood glucose levels reduced, and he was able to establish healthy routines, such as increase physical exercise and decrease overeating. In case 2, the participant’s fear of hypoglycemia greatly decreased, and she was able to confront many of her avoided situations and increase necessary visits to her diabetes clinic. Treatment satisfaction was high, and no adverse events were reported.

**Conclusions:**

It is possible to deliver a cognitive behavioral therapy intervention aimed at problem areas in diabetes online. Problem solving appears to help with problems in everyday routines and lifestyle choices. Exposure to aversive stimuli appears to be a plausible intervention specifically aimed at the fear of hypoglycemia. Larger and controlled studies are needed.

## Introduction

### Background

Type 1 diabetes mellitus (T1DM) is a chronic condition caused by deterioration in the ability of the pancreas to produce insulin, leading to insufficient insulin levels [1]. Lifestyle factors have not been found to increase the risk of T1DM, although, for example, diet and exercise are important to keep blood glucose stable when the disease is present [2]. Thus, patient self-care behaviors are necessary to maintain a stable and healthy blood glucose level [2]. People with T1DM are required to monitor their blood glucose levels multiple times per day and to plan and adjust insulin doses based on several factors. These factors include daily activities, food intake, exercise, ongoing infection, stress, and others, requiring well-functioning planning and problem-solving skills [3]. Fear of hypoglycemia is a fear specific to insulin-treated diabetes, which can lead to several unhelpful avoidance behaviors [4]. These behaviors generally include keeping blood glucose levels unhealthily high to try to avoid hypoglycemia, as well as avoiding places or situations viewed as potentially dangerous [5].

Psychological interventions aimed at people with T1DM have usually involved different types of cognitive behavioral therapy (CBT), often aimed at emotional coping and behavioral change [4]. One intervention that has been used for people with T1DM is problem solving, which is a technique that teaches a structured approach to solving practical and emotional problems [6]. Another CBT intervention that has been suggested is exposure, an often used and effective treatment component for anxiety disorders. This technique is used to confront feared stimuli, in order to decrease fear and to decrease avoidance of fearful situations [7]. For T1DM, exposure has mainly been proposed as an intervention for fear of hypoglycemia [4]. Exposure has previously been successfully tried for fear of hypoglycemia in a case study of face-to-face CBT [8]. Further evaluation of exposure has been proposed [4]. Exposure has been used successfully in guided self-help treatments for somatic conditions, including irritable bowel syndrome (IBS) [9], atrial fibrillation [10], and asthma [11].

Exposure may theoretically be a sound approach for people with T1DM who display avoidance behaviors and fear of symptoms, and this approach has been successful for similar chronic conditions. Regardless of the helpfulness of CBT in people with T1DM, access to psychologists with relevant knowledge of the diabetes population is severely limited [12]. Over several years, great progress has been made with CBT over the internet. Internet-delivered CBT (internet CBT) has some advantages over traditional CBT. Internet CBT requires less time per therapist and patient, and therefore allows more people to be treated. The format also means that the treatment can be available for people living in rural areas. The effects of internet CBT have been evaluated for a large number of conditions, generally with equal effects as traditional CBT [13]. One recent meta-analysis found 25 randomized controlled trials of CBT for diabetes, although only 5 of these were aimed at type 1 diabetes. The analysis suggested that CBT is an effective intervention to improve blood glucose and quality of life. However, none of the approaches had been completely delivered via the internet, and none had exposure as the main intervention [14]. There is some evidence suggesting the effectiveness of CBT for improving blood glucose and mental health in people with T1DM [14]. Therefore, we believe it is valuable to examine if online CBT for T1DM, including exposure, can be feasible, using a case study as the first step.

### Objective

The objective was to develop a manualized CBT treatment delivered online. In this study, we tested a treatment in the very early stage. The participants’ course of treatment was explored to get an early indication of what might and might not be practical in this type of treatment. To illustrate this, we describe 2 cases involving 2 participants with T1DM, who completed online guided self-help CBT. The illustrative cases will be used to revise and further adapt the treatment protocol adopted.

## Methods

### Participants

The details of the below 2 cases have been altered to protect the anonymity of the participants while keeping the functional similarity of the participants’ behavioral analysis and the characteristics of the treatment. The participants were recruited via advertisements on social media. They had to have a self-reported T1DM diagnosis, be unsatisfied with their blood glucose, or report other psychological or behavioral problems connected to their diabetes. In telephone interviews, the participants were asked about their diabetes history. The participants were asked about problematic areas with diabetes self-care and life in general. In particular, the participants were asked about specific emotional and behavioral reactions to problematic situations. The interviews were not recorded. See Table 1 for the timeline of the intervention.

**Table 1:** Timeline of the intervention.

Participant	Assessment	Treatment start	Treatment end
Participant 1 (Mr A)	January 25, 2018	March 06, 2018	April 27, 2018
Participant 2 (Ms B)	April 18, 2018	May 01, 2018	June 23, 2018

#### Case 1

Mr A was a man in his 40s with a university education. He had self-reported lifestyle problems and an increasing worry about complications. Mr A had found it difficult to engage in physical activity, which he knew was important to his health. He reported that he was very preoccupied with work and that it was challenging to fit physical activity into his schedule. He also found that he ate too much, reporting feelings of hunger even after he had eaten portions of adequate size according to the recommendations of his dietician. Mr A was already experiencing a number of diabetes-related complications (some mild and transient, and others moderately serious and permanent). He reported that he was often preoccupied with worry that the complications would deteriorate and that he would develop additional complications. He reported that it was problematic and a burden to plan the day with consideration of the disease, as well as find time and practical opportunity for self-care activities within daily life. Perfectionistic tendencies were another area that could cause problems, leading to unhelpful thoughts that if Mr A was not able to manage his diabetes perfectly, he might as well give in completely. This could lead to hopelessness and engaging in problematic behaviors, such as eating unhealthy food, reasoning that it did not matter anyway. Worry distracted him from engaging in more enjoyable activities, and could lead to a feeling of helplessness, as well as frustration and anger with the disease, or feeling overwhelmed.

Mr A reported that he had a general idea that he needed to change his behaviors and habits to improve his well-being. However, he had difficulties knowing precisely where to begin, and felt that he needed a structured way forward to fit self-care behaviors into his busy life. Previous approaches to behavioral change had also been hindered by unhelpful thoughts, worry, and perfectionism. We believed that problem solving and exposure would be helpful interventions for Mr A, as this would help him to structure what he already had in mind and help him go against unhelpful emotions to engage in behaviors he felt he needed.

#### Case 2

Ms B was a woman in her 30s with a university education. She reported high blood glucose levels and fear of hypoglycemia throughout several years. Her fears included worrying about losing consciousness or feeling humiliated in public, losing control, not having anyone there to help her, making serious mistakes, and becoming aggressive toward other people. A particular fear was to experience hypoglycemia during the night, which Ms B felt was a very frightening experience, sharing many characteristics of a panic attack. Furthermore, she reported that she had neglected her medical self-care to some degree when newly diagnosed in her early teens, acting as the disease would simply go away, although she knew that was not a realistic judgement. Stress was a reported problem area, which was accompanied by higher blood glucose. Ms B reported that her blood glucose level was less stable and that she did not find the time for proper self-care when under stress. This could lead to stressful thoughts about her diabetes, increasing stress in a vicious circle. A particularly unhelpful behavior was to eat to keep the blood glucose level high, in order to avoid hypoglycemic episodes during the night. This behavior would ease the fear and anxiety in the short term. In the long run, however, this led to hyperglycemia, with its unpleasant symptoms, such as thirst and fatigue. Likewise, Ms B preferred her blood glucose level to be on the higher side in other situations, such as when attending parties and driving. Another problematic area was that Ms B was afraid of diabetic health care itself. She avoided contacting her hospital diabetes unit as much as possible and reported aversive events where she had been educated in great detail about possible complications. Although the physician had all the best intentions, Ms B reacted with fear. Further mentions of complications made her anxious, and she wished to avoid the subject altogether. Ms B had been in contact with a psychologist about her diabetes before, but she was not satisfied with the intervention, reporting that they mainly talked in general, and that she was told to practice positive thinking, rather than behavioral interventions aimed at specific targets.

Ms B reported that her situation was beginning to be difficult to handle and felt overwhelmed by her fears. Our behavioral analysis showed that her fear had led to many avoidance and safety behaviors, with a negative impact on her blood glucose and general well-being. We hypothesized that it would be useful to have a particular focus on Ms B’s fear of hypoglycemia during the treatment. According to our hypothesis, if her fear of hypoglycemia was improved, it would lead to improvement overall.

### Ethical Considerations

The exercises in the treatment were discussed with a consultant endocrinologist and were considered safe. All procedures in the study were in accordance with the ethical standards of the regional ethics committee and with the 1964 Helsinki Declaration and its later amendments or comparable ethical standards. It was approved on January 04, 2018, by the regional authority Etikprövningsnämnden Stockholm (2017/2278-31). An addendum was approved by the same regional authority on February 12, 2018 (2018/300-32). Another addendum was approved on April 30, 2021, by the Swedish national authority Etikprövningsmyndigheten (2021-02263). Participants provided written informed consent for participation and publication.

### Measurements

This study relied on self-reported measures. The measures were filled out as online questionnaires, before and after treatment, and every week in some cases.

#### Blood Glucose

Glycated hemoglobin (HbA_1c_) is a well-used biomarker for the blood glucose level over several months [2]. Participants reported their latest known HbA_1c_ value and were encouraged to get a new measurement toward the end of treatment. In what way and how often participants measured their blood glucose level as part of their regular self-care was not controlled for in this study.

#### Problem Areas in Diabetes

The Problem Areas in Diabetes (PAID) Scale [15] is a scale used to assess areas of diabetes a patient may need guidance for from health care, and to screen for “emotional distress” in patient with diabetes. The PAID Scale consists of 20 items on a scale from 0 (“not a problem”) to 4 (“serious problem”). The total score ranges from 0 to 100, and scores over 40 indicate “emotional distress.” According to the authors of the scale, people with a score of 40 or higher require attention from their diabetes team, and the score is expected to drop 10-15 points following medical and educational interventions. The scale has been validated and shown to be acceptable in a diabetes population [15]. This scale was measured weekly.

#### Fear of Hypoglycemia

The Hypoglycemia Fear Survey (HFS) [16] was developed to measure to what degree a patient with diabetes is afraid of hypoglycemia. This scale explores avoidance behaviors and worry in relation to hypoglycemia. It has 23 items with a range from 0 (“never”) to 5 (“always”). The total score is 0-92, with a mean score of 25 in a clinical sample. This scale was measured weekly.

#### Anxiety

Generalized Anxiety Disorder-7 (GAD-7) [17] is commonly used to measure anxiety. It consists of 7 items using a scale from 0 (“not at all”) to 3 (“nearly every day”). The total score is 0-21 points. The authors suggest cutoff scores for mild (5), moderate (10), and severe (15) anxiety. The scale has been found to be valid and reliable [17].

#### Depression

The Patient Health Questionnaire (PHQ-9) [18] consists of 9 items on a scale from 0 (“not at all”) to 3 (“nearly every day”), with a total score of 0-27. The authors suggest a cutoff at 5 (possible depression) and 15 (probable depression). The scale has been found to be valid and reliable [18].

#### Stress Reactivity

The perceived stress scale [19] consists of 14 items on a scale from 0 (“never”) to 4 (“very often”), with a total score of 0-40. The authors suggest intervals, with 0-13 indicating low stress, 14-26 indicating moderate stress, and 27-40 indicating high perceived stress. The scale has been shown to be valid and reliable [19].

#### Credibility/Expectancy

Participants’ perception of the credibility of the treatment and their expectation of the final result were assessed with the Credibility/Expectancy Questionnaire (CEQ) [20], using a scale from 1 to 6, with a total score of 0-50. Higher scores suggest better credibility/expectancy. The scale has been found to be valid and reliable. The scale was administered 1 week into treatment.

#### Treatment Satisfaction

The Client Satisfaction Questionnaire (CSQ) [21] consists of 8 items on a scale from 1 to 4, with a total score of 8-32. The score can be recalculated with a total score from 25 to 100. A higher score suggests a higher satisfaction [21].

#### Adverse Events

Participants answered questions about whether they had experienced any adverse events, and if so, they were told to elaborate on these experiences. The questionnaire was unpublished.

#### Subjective Assessment

Participants were asked about subjective blood glucose improvement, using the Subjective Assessment Questionnaire (SAQ), with a 6-point scale ranging from “much declined” to “much improved.” This type of scale is often used for other somatic conditions and has been found to be useful and valid [22].

### Therapeutic Intervention

The treatment was delivered entirely via the internet, through a secure treatment platform. Participants received homework each week, answering questions about the psychoeducation and doing exercises in their everyday life. Participants received psychoeducation based on material from a previously evaluated CBT group treatment for T1DM [23] and adapted material from an internet-delivered treatment for IBS [9]. The material from the IBS treatment was general information on worry about symptoms and exposure to avoided stimuli, which was easily adapted for T1DM by changing examples. The participants had access to 5 modules of psychoeducation, each approximately corresponding to 10 written A4 pages. The active treatment consisted of established CBT interventions. The main components used were problem solving and exposure. There were also some cognitive interventions, namely information about thinking traps and basic cognitive restructuring, and additional interventions such as assertiveness training and life values. The participants read the same material and tried all interventions. After about half of the treatment, a main focus was chosen according to their specific problem in a discussion with the therapist.

#### Therapist and Supervisor

The therapist (DK) was a master’s student at the time of the study and was trained in CBT. His supervisor for the master’s thesis (BL) also acted as a therapy supervisor. The supervisor is a professor of psychology and a licensed psychologist specialized in CBT. The therapist could be reached through text messages in the online platform at any time throughout the treatment. The role of the therapist was to provide guidance and support, provide feedback, and answer questions. The therapist also gave access to the next module after the previous one was completed. Study participants were notified by an automatic text message each time the therapist had made contact via the online platform. If participants were inactive, they were encouraged to continue their assignments via text messages or phone calls.

#### CBT Model

**Figure 1:**
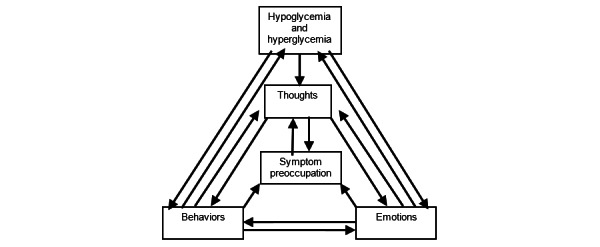
General cognitive behavioral therapy model. Adapted from T Anderbro and Susanne Amsberg (unpublished data, 2004) and B Ljótsson and E Hedman-Lagerlöf (unpublished data, 2012).

**Figure 2:**
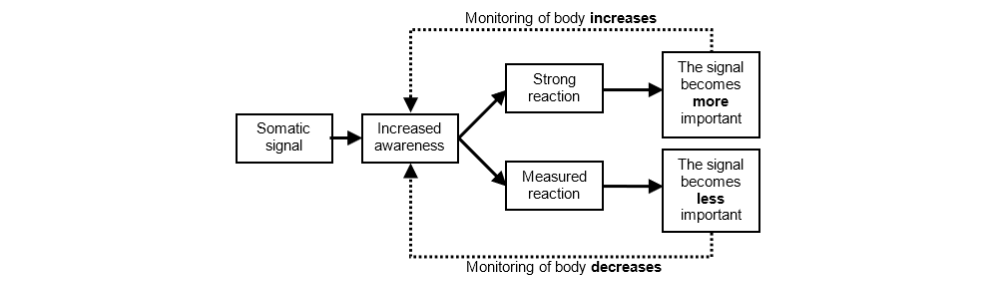
Suggested exposure model for fear of hypoglycemia. Adapted from B Ljótsson and E Hedman-Lagerlöf (unpublished data, 2012).

We mainly used a general cognitive-behavioral model, where we assumed that thoughts, emotions, and behaviors interact and influence each other, which was largely influenced by an exposure-based CBT program for IBS [9]. See Figures 1 and 2 for an overview. This model also assumes that these influence blood glucose levels directly via behaviors or indirectly via thoughts and emotions that influence behaviors. In some cases, emotions, such as stress, may influence general mood, which may directly influence blood glucose, as suggested by a CBT group treatment for T1DM [4]. Increased awareness may lead to symptom preoccupation, making a person more observant of potential symptoms [9]. We worked from an exposure-based paradigm, where we assumed that humans avoid unwanted experiences that may be aversive short term, but still are important and helpful [9]. Exposing oneself to aversive stimuli or situations decreases anxiety over time, most likely due to extinction of fear [7]. It is possible that symptoms of anxiety can overlap with symptoms of low blood glucose, thus conflating the two [4]. Exposing oneself to feared situations in the presence of anxiety symptoms should, according to this model, decrease fear of hypoglycemic symptoms, and decrease avoidance of feared situations or situations where low blood glucose levels would be unwanted.

The intervention also contained a few approaches that were not necessarily based on this model but did not contradict it, including cognitive restructuring, assertiveness communication skills, and life values. These were components from the previously evaluated CBT group treatment on which this intervention was partially based [4]. These were kept in addition to the main exposure-based model, in order to explore if these could be useful complementary interventions.

### Content of Treatment Modules

#### Module 1

The module began with practicalities followed by general education on T1DM. The module also introduced the CBT model and education of general CBT principles. Participants were taught about our proposed interaction of thoughts, emotions, and behaviors. This, in turn, would influence self-care and blood glucose levels. Participants were introduced to the concept of functional behavioral analysis and learned how to identify what prevented them from behaving in accordance with their goals.

#### Module 2

The module continued the psychoeducation with a focus on stress and negative emotions. The intervention *problem solving* was introduced and presented mainly as an aid for self-care. This was presented as being particularly useful during periods of stress or negative mood, as sometimes self-care behaviors decrease in priority. The potential impacts of negative thoughts, as well as how to identify them were also taught. The participants identified any negative thoughts on their own and discussed how these might interfere with their everyday life, with a focus on diabetes.

#### Module 3

The module contained psychoeducation about anxiety and worry, and the roles these play in unhelpful behaviors. The participants were educated about the concept of exposure to aversive stimuli, and were provided further information on how to facilitate behavioral change. This module also introduced life values, often used in acceptance and commitment therapy [24], as an additional intervention. The participants considered the various areas in their own life where diabetes could have interfered. The participants were also encouraged to plan and work toward better integrating diabetes self-care into the life they wished to lead. As an additional skill, the participants were taught about assertiveness training.

#### Module 4

The module was mainly dedicated to exposure exercises. These were based on the problematic behaviors and situations participants had analyzed earlier in treatment. With the help of their therapist, the participants constructed an exposure hierarchy and used that to plan exposure exercises. If exposure to aversive stimuli was not appropriate, the participant would continue to work with behavioral change, for instance, with the help of problem solving and other helpful skills previously taught.

#### Modules 5-7

There was no additional information at this point in treatment. The participants continued to practice and were guided by their therapist as earlier. If a participant had a slower pace than 1 module per week, this could be a time to catch up.

#### Module 8

In the final module, the participants constructed a maintenance plan, based on the skills they used the most.

## Results

### Overview

The overall results in the 2 cases are presented in Table 2.

**Table 2:** Results in the 2 cases.

Variable	Participant 1 (Mr A)			Participant 2 (Ms B)	
	Pretreatment	Posttreatment	Pretreatment		Posttreatment
HbA_1c_^a^ (mmol/mol)	60	50	62		—^b^
PAID^c^ Scale score	50	28	92		53
HFS^d^ score	8	7	64		40
GAD-7^e^ score	4	4	12		13
PHQ-9^f^ score	8	4	8		18
PSS^g^ score	32	16	35		34
SAQ^h^ score	N/A^i^	6	N/A		4
AEs^j^, n	N/A	0	N/A		0
CSQ^k^ score	N/A	75	N/A		100

^a^HbA_1c_: glycated hemoglobin.

^b^Not reported.

^c^PAID: Problem Areas in Diabetes.

^d^HFS: Hypoglycemia Fear Scale.

^e^GAD-7: Generalized Anxiety Disorder-7.

^f^PHQ-9: Patient Health Questionnaire-9.

^g^PSS: Perceived Stress Scale.

^h^SAQ: Subjective Assessment Questionnaire.

^i^N/A: not applicable.

^j^AEs: adverse events.

^k^CSQ: Client Satisfaction Questionnaire.

### Case 1

Problem solving was the primary intervention for Mr A. He was able to try out solutions to numerous problems. For example, he managed to fit squash into his schedule, and had a goal to play once a week with his friends and to walk 8000-12,000 steps per day, in order to get an adequate amount of daily exercise. He made a rule to only take one portion of food for each meal, and he found it effective to go against his worry to do these physical activities, even though they reminded him of his complications. This could possibly be considered a form of exposure to aversive stimuli. Before treatment, Mr A had a score of 50 in the problem areas on the diabetes scale. After treatment, the score had dropped to 28, meaning a decrease of 22 points. Answering a postmeasurement question, Mr A stated that his blood glucose greatly improved. Another improvement was clinically significantly decreased stress according to the perceived stress scale, with a score of 32 before treatment and 16 after treatment. Fear of hypoglycemia did not seem to be a problematic area for this participant, beginning with a low grade of 8 points compared to an average of 25 in a clinical sample [4]. The value increased somewhat to about 20 through the course of treatment, but gradually decreased again to a score of 7 after treatment. Mr A reported 75 out of 100 points in the CSQ, indicating a high satisfaction with treatment. He stated that he was very satisfied with the treatment overall. Mr A also gave feedback on the psychoeducation material, feeling that the material was too long, and he wished for it to be more concise and to the point.

After treatment, Mr A reported his long-term blood glucose levels to us, which showed a decrease in HbA_1c_ from 60 to 50 mmol/mol, which is not only a clinically significant change in the desired direction, but also within the general goal value of <52 mmol/mol in Swedish diabetes care [25]. Mr A reported that it was the best HbA_1c_ value in his clinical history and that he attributed this to the treatment. It is important to remember that a decrease in HbA_1c_ does not necessarily equal a healthier lifestyle but could be a misleading finding because of repeated hypoglycemic episodes. This was not the case for this participant, however, as neither increased frequency of hypoglycemia nor any other adverse events were reported upon questioning. There were still some hyperglycemic episodes, meaning that there was room for continued decrease in hyperglycemia, but the blood glucose levels were quite satisfactory overall, according to the Swedish guidelines.

Overall, considering Mr A’s satisfaction with the treatment, the decreased problematic areas associated with his diabetes condition, and the decreased blood glucose levels, the treatment appeared to be successful.

### Case 2

Ms B’s treatment consisted mainly of exposure exercises aimed at her fear of hypoglycemia and associated avoidance behaviors. Another important area covered was her fear of visiting the diabetes hospital unit, which could be considered exposure to the emotion of shame, as hospital visits reminded her of her perceived failures as a patient with diabetes. She gradually approached health care, attending a lecture at her diabetes clinic about complications and their prevention. She reported that this increased her anxiety severely, but she was able to stay in the situation until it reduced. As a result of this, Ms B reported that she was able to form a better relationship with her diabetes care staff, which could prove beneficial to her future health. Another important area was the safety behavior of eating extra food before bedtime to decrease the risk of night-time hypoglycemia. Ms B gradually decreased the amount she ate and was eventually able to overcome this safety behavior, with potentially significant results for her overall blood glucose levels. Initially, she reported increased anxiety and difficulty falling asleep. She also reported waking in the middle of the night, feeling anxious, and checking her blood glucose levels. After less than a week, however, she reported that her anxiety started to subside and that she was able to sleep uninterrupted again. Eventually, she was able to stop the safety behavior of excessive eating before bedtime.

Ms B reported a significant decrease in diabetes-specific problems, with a decrease from 92 to 53 on the PAID Scale. This is still a high number, but the decrease was much greater than the expected decrease after education only, according to the authors of the scale. Relevant to her main problems, Ms B reported a significantly lower fear of hypoglycemia of 40 points on the hypoglycemia fear scale compared with 64 points before treatment, which was a clinically significant decrease, and she rated her blood glucose levels as somewhat improved. Ms B reported a score of 100 out of 100 points on the client satisfaction scale, indicating excellent treatment satisfaction. Unfortunately, we did not know if the treatment had any effects on Ms B’s blood glucose or HbA_1c_ levels, as she did not report a posttreatment HbA_1c_ value. Nevertheless, we consider the treatment successful because the treatment was able to target her fear of hypoglycemia using exposure to feared stimuli. It should be noted that her depression rating increased after the treatment, as measured by the patient health questionnaire. When asked, she did not think that this was a negative effect of the intervention but attributed her decreased mood to external factors.

### Adverse Events

No adverse events or other unexpected events were reported in either of the cases.

## Discussion

### Summary

In this case study, the online guided self-help CBT treatment of 2 participants has been described. The aim was to test an early version of this treatment, in order to revise and improve the protocol. The 2 participants were treated using the same treatment manual, and they read every module and tried all interventions. However, they both had a personalized focus. In case 1, the participant Mr A chose to focus on problem solving in the later part of the treatment. After treatment, he showed improved blood glucose and other relevant improvements. In case 2, the participant Ms B. chose to focus on exposure in the later part of treatment. She showed a significant decrease in her fear of hypoglycemia and other relevant improvements.

### Principal Findings

In the 2 cases, participants had favorable outcomes on their most relevant measurements. The participants completed all modules and were highly satisfied with their treatment. Mr A felt mainly helped by problem solving, which is logical, as this intervention was most suitable to help him accomplish the lifestyle changes desired. Conversely, Mr A found it difficult and redundant to construct exposure exercises, as he did not consider himself to be driven by fear or anxiety. Instead, he preferred to focus on structured problem solving. We believe, however, that there were some elements of exposure to other avoided emotions involved, such as worry and discomfort. Ms B focused on exposure for her fear of hypoglycemia, which was much improved, as well as her general problem areas in diabetes.

### Limitations

The results from this case study must be carefully considered as there are limitations. This is a report on only 2 cases, and the participants may have had personal characteristics and environmental factors that could explain their improvements. Furthermore, we were not able to control for attention. We do not know if their improvements are due to the treatment or the fact that someone kept a watchful eye on them. In fact, Mr A stated that he partially attributed attention from the therapist as a factor for his improvement. Finally, we did not receive a report on Ms B’s blood glucose level. This is significant missing data, which we have no easy way of retrieving.

### Comparison With Prior Work

To our knowledge, this is the first study that tentatively explored an online CBT intervention with an exposure model for T1DM. The outcomes are consistent with those in earlier studies on CBT and T1DM, as CBT has been shown to improve blood glucose and mental health [4,12]. The experiences of these participants indicate that a CBT treatment with multiple interventions may be useful. For example, an entirely exposure-focused treatment would have been less relevant for Mr A. The results of Ms B are in line with the findings in few previous studies that have evaluated exposure to aversive stimuli as an intervention for fear of hypoglycemia [4,8].

### Conclusions

These cases provide some insights on how an online-delivered T1DM CBT program could look, and preliminarily suggest that the use of both problem solving and exposure could be useful in CBT for T1DM. In accordance with the feedback of Mr A, the treatment can be further adapted to decrease the amount of text and introduce learning exercises through examples. These changes may improve the treatment and make the information focused and relatable. Looking forward, we suggest a feasibility study with a larger group of participants, to examine the safety, acceptability, and approach of the intervention, as well as its preliminary effects.

### Patient Perspective

Mr A was very satisfied with his experience overall and felt that his condition was much improved. As mentioned, he had some critique of the education material provided, but felt that the contact with the therapist made up for it. Ms B was very satisfied and felt that her condition was much improved. She especially appreciated the applied and concrete nature of CBT.

## Abbreviations

CBT: cognitive behavioral therapy
CSQ: Client Satisfaction Questionnaire
HbA_1c_: glycated hemoglobin
IBS: irritable bowel syndrome
PAID: Problem Areas in Diabetes
T1DM: type 1 diabetes mellitus
